# EXPLORING FACILITATORS AND BARRIERS TO CONDUCTING SPIRITUAL HISTORIES IN OLDER ADULTS

**DOI:** 10.1093/geroni/igad104.0932

**Published:** 2023-12-21

**Authors:** Monica Beck, Amelia Gallagher

**Affiliations:** University of Alabama in Huntsville, Huntsville, Alabama, United States; The University of Alabama in Huntsville, Huntsville, Alabama, United States

## Abstract

Nurses can relieve spiritual suffering in older adult cancer patients through spiritual histories, yet may be reticent to engage in spiritual conversations. The qualitative aim of Lift the Spirit, a pilot feasibility and acceptability study, was to explore factors affecting cancer nurses’ perceptions of the barriers and facilitators to conducting spiritual histories in clinical practice. Lift the Spirit, an enhanced educational communication intervention, was designed to equip nurses with the knowledge, skills, and attitudes to conduct spiritual histories. A purposive sample of nurses (n = 17) independently engaged in an online multimedia course, Spiritual Assessment in Clinical Practice, then synchronously role-played conducting a spiritual history in dyads (nurse/patient), engaged in a skills performance, and participated in Debrief Interviews. Qualitative data were collected during Debriefing Interviews using the Debrief Interview Guide. Interpretive description guided the qualitative content analysis of Debrief Interview data. Structural coding was used to sort data into barriers and facilitators on the first pass. Next, within each structural code, data fitting the definition of barrier and facilitators were inductively descriptively coded. Participants described barriers and facilitators at the levels of institution/profession (lack of education and training), self (vulnerability, perceived riskiness), and patient (cultural difference) that were similar to barriers already noted in the literature. Facilitators included feeling equipped and supported and having external cues as reminders. The degree of angst, fear, and vulnerability expressed towards conducting spiritual histories was surprising. Equipping nurses with the knowledge, skills, and attitudes is essential to reduce spiritual suffering in older adults.

